# Draft Genome Sequence of *Staphylococcus simulans* UMC-CNS-990, Isolated from a Case of Chronic Bovine Mastitis

**DOI:** 10.1128/genomeA.01037-13

**Published:** 2013-12-12

**Authors:** Michael J. Calcutt, Mark F. Foecking, Hsin-Yeh Hsieh, Jeanette Perry, George C. Stewart, John R. Middleton

**Affiliations:** Department of Veterinary Pathobiology, University of Missouri, Columbia, Missouri, USAa; Bond Life Science Center, University of Missouri, Columbia, Missouri, USAb; Department of Veterinary Medicine and Surgery, University of Missouri, Columbia, Missouri, USAc

## Abstract

Coagulase-negative staphylococci are frequently isolated from cases of subclinical bovine mastitis. Reported here is a draft genome sequence of *Staphylococcus simulans* UMC-CNS-990, an isolate recovered from a chronic intramammary infection of a Holstein cow. Unexpectedly, a cluster of genes encoding gas vesicle proteins was found within the 2,755-kb genome.

## GENOME ANNOUNCEMENT

Relatively little is known about the pathogenesis and niche adaptation of coagulase-negative staphylococci that cause bovine mastitis (1). Here, we report the determination of a draft genome sequence of *Staphylococcus simulans* UMC-CNS-990. This strain was isolated in September 2005 from milk of the right rear mammary quarter of a Holstein cow at the University of Missouri Dairy (under IACUC approval). The intramammary infection persisted, based on monthly milk sampling, until February 2006 and was associated with a geometric mean milk somatic cell count of 185,000 cells/ml. Isolate UMC-CNS-990 and the isolate recovered in February 2006 had identical SmaI pulsotypes.

Genome sequencing was performed by the Genome Institute at Washington University, St. Louis, MO. Fragment library sequencing (454 titanium chemistry) resulted in 242,529 reads that assembled into 40 contigs using Newbler (Roche). The draft genome of 2,755,177 bp was sequenced to a 33-fold depth and comprised 35.85% G+C. The largest contig was 352,956 bp and the contig *N*_50_ was 252 kb. Auto-annotation of the contigs was performed at the National Center for Biotechnology Information using the PGAP pipeline with 2,605 open reading frames (ORFs) annotated using GeneMarkS+. Analysis of rRNA encoding genes predicted the presence of 5 rRNA operons; 58 tRNA genes were identified.

During the course of this project, the genome sequence of *S. simulans* strain ACS-120-V-Sch, a human vaginal isolate and reference genome in the Human Microbiome project (2), became available for comparative analysis. Predictably, large regions of contigs exhibited extensive colinearity; instances wherein the synteny was significantly punctuated coincided with integration of strain-variable prophage-like elements. Clustered regularly interspaced short palindromic repeat (CRISPR) units with different spacer regions were identified in both strains.

One unanticipated feature of the *S. simulans* genomes is an approximately 6-kb chromosomal locus carrying genes for putative gas vesicle proteins (3). This *gvp* cluster is located at the same chromosomal site in strain ACS-120-V-Sch but is not present in any sequenced genome from the *Staphylococcaceae*. This sporadically distributed locus thus represents a novel component of the pangenome of these taxa.

Three plasmid sequences were identified in strain UMC-CNS-990: two small cryptic replicons (3.54 kb and 4.13 kb), together with a large, nonconjugative plasmid of ~60 kb. The latter encodes (i) a novel variant of the Vga(A)_LC_ resistance determinant (4); (ii) all 8 incursions of IS*431* in the *S. simulans* genome; (iii) tandem genes encoding small peptides with characteristic signatures of the lantibiotic SP_1948 family (5), located between genes for lantibiotic processing, cyclization, and export; and (iv) a gene cluster exhibiting >99% identity with the *nukTFEGH* genes that are involved in biosynthesis and immunity to the lantibiotic nukacin ISK-1 (6). However, the *nukA* structural gene is not present and *nukT* is truncated, possibly due to an adjacent IS*431* unit*.*

The genome sequence data reported here are the first from an *S. simulans* isolate linked to animal disease and should prove a useful adjunct to studies of gene distribution patterns and pathogenomics of coagulase-negative staphylococci.

### Nucleotide sequence accession numbers.

This whole-genome shotgun project has been deposited at DDBJ/EMBL/GenBank under the accession number AXDY00000000. The version described in this paper is version AXDY01000000.
